# Fatal Trichosporon asahii Fungemia Following Polymicrobial Bacteremia in a Non-neutropenic Elderly Patient: A Case Report

**DOI:** 10.7759/cureus.89531

**Published:** 2025-08-07

**Authors:** Yuko Fujikawa, Yoshihisa Katsuta, Yoshiko Shibata, Kenjiro Akai, Katsuhiko Kamei

**Affiliations:** 1 Department of Internal Medicine, Ishinomaki Municipal Hospital, Ishinomaki, JPN; 2 Medical Mycology Research Center, Chiba University, Chiba, JPN

**Keywords:** diabetes, elderly, fungemia, non-neutropenic, trichosporonosis, voriconazole

## Abstract

*Trichosporon *species are known to cause disseminated infections in immunocompromised hosts, typically in patients with hematological malignancies undergoing chemotherapy and those with a history of antifungal use. This case report described a non-neutropenic 85-year-old male patient with *Trichosporon asahii *fungemia following polymicrobial bacteremia. He presented with fever and disturbed consciousness and was admitted for sepsis (day 1). Blood cultures tested positive for *Enterobacter cloacae*, *Klebsiella oxytoca*, *Enterococcus faecalis*, and Methicillin-resistant *Staphylococcus aureus* (MRSA). Sepsis improved with the administration of piperacillin-tazobactam and vancomycin. Blood culture on day 14 was negative for bacteria but positive for yeast-like organisms, which were identified as *Trichosporon asahii *by mass spectrometry. After voriconazole administration, the blood culture became negative. However, the echocardiograph suggested infectious endocarditis, and vertebral magnetic resonance imaging (MRI) suggested osteomyelitis and epidural abscess of the lumbar spine. Despite long-term antifungal and antibacterial treatments, the patient’s general condition deteriorated, and he died of aspiration pneumonia on day 119. Multiple factors, including age, diabetes mellitus, malnutrition, combined with polymicrobial bacteremia, and antibacterial usage, contributed to *T. asahii* fungemia. *T. asahii* may have invaded the systemic bloodstreamthrough damaged intestinal mucosal tissues. Trichosporonosis has emerged in non-neutropenic individuals. Further investigations of invasive non-*Candida* fungal infections in this population are required.

## Introduction

*Trichosporon* species are yeast-like organisms commonly found in nature, such as in soil, and are part of the normal flora of human skin and the gastrointestinal tract. They are recognized as opportunistic pathogens that cause invasive and often fatal infections known as trichosporonosis, mostly in immunocompromised individuals. *Trichosporon asahii* is the major species responsible for trichosporonosis [1,2]. Trichosporonosis often presents with fungemia and disseminated infections involving the skin, lungs, liver, spleen, and central nervous system [3], with mortality ranging from 30% to 90% [4]. The major risk factor for trichosporonosis is neutropenia, particularly in patients with hematological malignancies and undergoing chemotherapy [3,5]. Trichosporonosis often occurs as a breakthrough infection in patients receiving echinocandins or polyenes [6,7]. Other risk factors for trichosporonosis include the placement of central venous catheters, broad-spectrum antibiotic use [8], steroid use [9], and diabetes mellitus [10]. The suspected routes of infection are bloodstream infection through devices, particularly intravenous catheters, and translocation from the gastrointestinal tract through damaged mucosae [11], as well as damaged skin barriers in patients with severe burns [12], diabetic necrotic foot, or toxic epidermal necrolysis.

Voriconazole or posaconazole is moderately recommended as first-line therapy for trichosporonosis, while fluconazole is a first-line alternative. Liposomal amphotericin B or amphotericin B deoxycholate is marginally recommended as second-line therapy. Two weeks of antifungal treatment after a negative blood culture is recommended for fungemia, and a longer duration of therapy is recommended if there is organ involvement [4].

## Case presentation

An 85-year-old male patient was admitted to our hospital with sepsis. He had a history of colon cancer surgery without a permanent colostomy and no recurrence. His medical history also included diabetes mellitus, hypertension, hyperuricemia, dementia, and urethral catheter placement for prostatic hypertrophy. The patient previously worked as a carpenter. He lived alone and utilized daycare facilities. The patient complained of appetite loss the previous day and was transported by ambulance to the hospital because of fever and disturbed consciousness (day 1). He scored 13 (E3V4M6) on the Glasgow coma scale. Vital sign measurements included body temperature of 38.6 ℃, blood pressure of 96/53 mmHg, heart rate of 103 bpm, and oxygen saturation of 90% while receiving 2 L/minute of oxygen through nasal cannula. Laboratory tests revealed elevated white blood cell and neutrophil counts, anemia, thrombocytopenia, elevated glucose and HbA1c levels, decreased serum protein and albumin levels, renal failure, coagulopathy, and bacteriuria (Table 1).

**Table 1:** Laboratory data on admission (day 1) and on day 14. Laboratory tests on admission (day 1) revealed elevated white blood cell and neutrophil counts, anemia, thrombocytopenia, elevated glucose and HbA1c levels, decreased serum protein and albumin levels, renal failure, coagulopathy, and bacteriuria. Blood and urine cultures were positive for multiple bacteria. On day 14, inflammatory signs were improving, but serum β-D-glucan was markedly elevated. Immunoglobulin levels were normal, and serum HIV and HTLV-1 antibody levels were negative. Blood cultures were negative for bacteria but positive for *Trichosporon asahii*. WBC, white blood cell count; RBC, red blood cell count; Hb, hemoglobin; Hct, hematocrit; MCV, mean corpuscular volume; Plt, platelet count; PT-INR, prothrombin time–international normalized ratio; APTT, activated partial thromboplastin time; LDH, lactate dehydrogenase; AST, aspartate aminotransferase; ALT, alanine aminotransferase; ALP, alkaline phosphatase; TP, total protein; Alb, albumin; BUN, blood urea nitrogen; Cre, creatinine; Na, sodium; K, potassium; Cl, chloride; Ca, calcium; P, phosphate; CRP, c-reactive protein; BNP, b-type natriuretic peptide; HbA1c, glycated hemoglobin; IgG, immunoglobulin G; IgA, immunoglobulin A; IgM, immunoglobulin M; HIV-Ab, human immunodeficiency virus antibody; HTLV-1 Ab, human T-cell leukemia virus type 1 antibody; SG, specific gravity; pro, protein; glu, glucose; ub, urobilinogen; bil, bilirubin; ket, ketones; ob, occult blood; wbc, white blood cells; nit, nitrite; RBC (/hpf), red blood cells per high-power field; WBC (/hpf), white blood cells per high-power field; MRSA, methicillin-resistant Staphylococcus aureus; O2, oxygen; BE, base excess

	Day 1	Day 14	Normal range
Blood cell count			
WBC (/μL)	17,490	10,060	4,500-9,000
Neut (%)	94	82.6	39-73
Eosino (%)	2.5	1.1	0-7
Baso (%)	0.2	0.3	0-3
Lymph (%)	2.6	13.1	18-51
Mono (%)	0.7	2.9	1-12
RBC (x10^6^/μL)	3.06	3.04	4.38-5.34
Hb (g/dL)	9.0	9.0	13.9-16.9
Hct (%)	27.1	26.2	40.1-48.6
MCV (fL)	88.6	86.2	84.7-98.1
Plt (x10^4^/μL)	7.6	10.7	13.2-38.0
Coagulation			
PT-INR	1.66		0.85-1.15
APTT (sec)	40.5		24-32
D-dimer (μg/mL)	49.4		0-1
Serum chemistry/serology			
T. Bil (mg/dL)	0.8	0.6	0.2-1.2
LDH: IFCC (U/L)	251	157	124-222
AST (U/L)	27	9	12-33
ALT (U/L)	8	6	5-35
ALP: IFCC (U/L)	85	76	38-113
TP (g/dL)	4.3		6.7-8.1
Alb (g/dL)	1.6		3.9-4.9
BUN (mg/dL)	36.6	51.9	8-20
Cre (mg/dL)	3.49	4.95	0.53-1.02
Na (mEq/L)	144	137	136-145
K (mEq/L)	3.5	3.4	3.4-4.5
Cl (mEq/L)	111	102	100-108
Ca (mg/dL)	7.3		8.6-10.1
P (mg/dL)	3.0		2.5-4.5
CRP (mg/dL)	11.75	3.63	0-0.3
procalcitonin (mg/dL)	134.65		0-0.05
BNP	1042.6		0-18.4
Glucose (mg/dL)	112		70-110
HbA1c (%)	8.9		4.6-6.2
IgG (mg/dL)		1545	870-1700
IgA (mg/dL)		580	110-410
IgM (mg/dL)		58	33-190
HIV-Ab		-	-
HTLV-1 Ab		-	-
β-D‐Glucan (pg/mL)		475	0-20
Cryptococcus Ag		-	-
Blood gas analysis (O2:2L/minute)			
pH	7.477		7.35-7.45
pCO2 (mmHg)	25.5		35-45
pO2 (mmHg)	58		75-
HCO3 (mEq/L)	18.9		23-28
BE (mEq/L)	-5		-2.2-1.2
Urinalysis			
pH	7.0		4.5-7.5
SG	1.015		1.005-1.030
pro	3+		-
glu	-		-
ub	+-		+-
bil	-		-
ket	-		-
ob	2+		-
wbc	4+		-
nit	-		-
RBC (/hpf)	5-9		-
WBC (/hpf)	>100		-
bacteria (cocci)	2+		-
bacteria (rods)	3+		-
Microbiology			
Blood culture	Enterobacter cloacae (2/2 sets)	Trichosporon asahii (2/2 sets)	
	Klebsiella oxytoca (2/2 sets)		
	Enterococcus faecalis (2/2 sets)		
	MRSA (1/2 sets)		
Urine culture	Pseudomonas aeruginosa (1.0 x 10^5^ cfu/mL)		
	MRSA (5.0 x 10^4 ^cfu/mL)		

**Figure 1:**
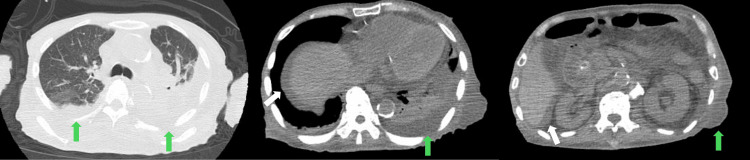
Chest and abdominal computed tomography (CT) on admission. CT showed bilateral pleural effusion (the green arrow on the left and middle panels), ascites (the white arrow on the middle and right panels), and subcutaneous edema (the green arrow on the right panel).

Brain computed tomography (CT) showed senile atrophy. Chest and abdominal CT demonstrated bilateral pleural effusion, ascites, and subcutaneous edema (Figure 1).

Blood cultures were positive for *Enterobacter cloacae*, *Klebsiella oxytoca*, *Enterococcus faecalis*, and MRSA, while urine cultures were positive for *Pseudomonas aeruginosa* and MRSA. Septic conditions improved with the administration of piperacillin-tazobactam and vancomycin. Blood cultures on day 14 were negative for bacteria but positive for yeast-like organisms, which were identified as* T. asahii* by mass spectrometry (MALDI Biotyper, score 2.02, library: BDAL Ver.11, MSPs: 10833 (MSP: Main Spectra)), with antifungal sensitivity by broth dilution method (Yeast-like fungus DP; Eiken Chemical, Tokyo, Japan), as shown in Table 2.

**Table 2:** Antifungal susceptibility of *Trichosporon asahii* detected from blood culture. AMPH-B, amphotericin B; 5-FC, flucytosine; MCZ, miconazole; FLCZ, fluconazole; ITCZ, itraconazole; MCFG, micafungin; VRCZ, voriconazole; CPFG, caspofungin

Antifungal agent	MIC (μg/mL)
AMPH-B	1
5-FC	8
MCZ	0.25
FLCZ	1
ITCZ	0.25
MCFG	>16
VRCZ	0.06
CPFG	8

**Figure 2:**
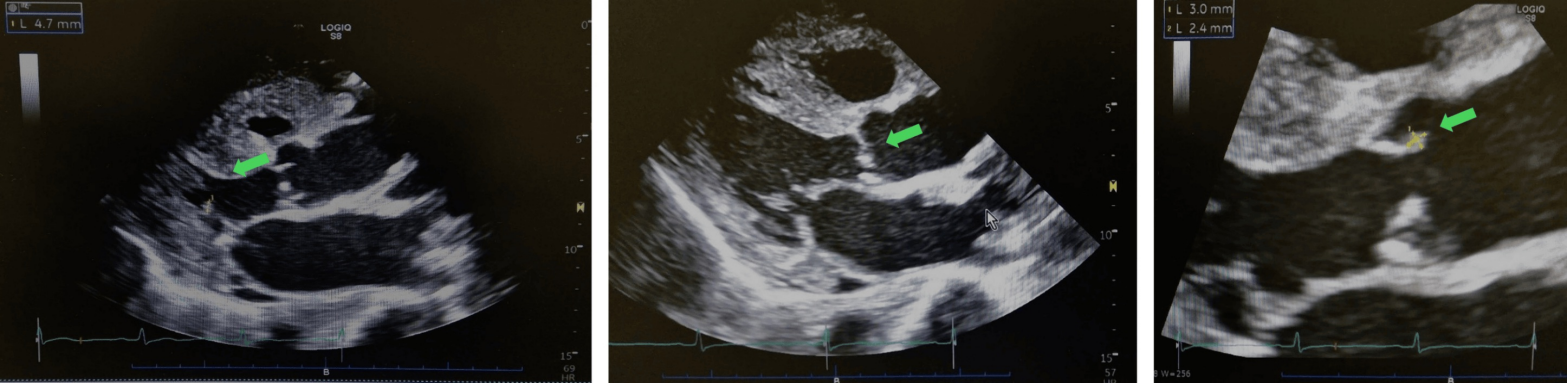
Echocardiograms taken on day 20 (left panel) and day 27 (right two panels). Echocardiogram on day 20 suggested vegetation of the mitral valve (the arrow on the left panel). It disappeared on day 27, but a new vegetation-like mobile mass of the right coronary cusp of the aortic valve was observed (the arrows on the middle and right panels).

Although inflammatory signs were improving, serum β-D-glucan was markedly elevated. Immunoglobulin levels were normal, and serum HIV and HTLV-1 antibody levels were negative (Table 1). Echocardiography revealed vegetation of the mitral valve, suggesting infectious endocarditis (the left panel of Figure 2).

**Figure 3:**
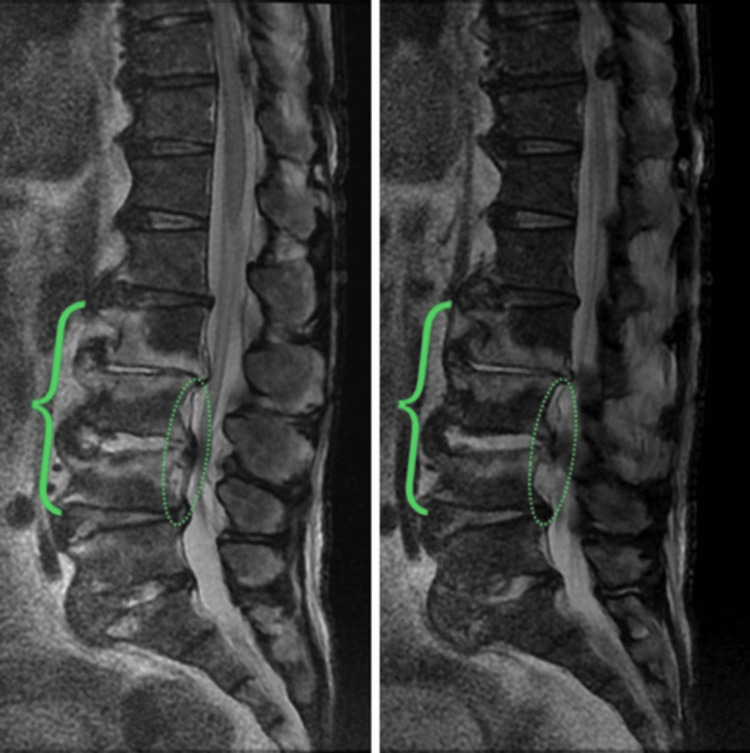
Vertebral magnetic resonance imaging (MRI) on day 25: T2-weighted image (T2, left) and T2-weighted image with frequency-selective fat suppression (T2FS, right). MRI T2 and T2FS showed high intensity of L2-L4 (the bracketed) vertebrae suggesting osteomyelitis and high intensity area posterior to the L3-L4 vertebrae (the circled areas) suggesting an epidural abscess.

Additionally, vertebral MRI suggested osteomyelitis and an epidural abscess of the lumbar spine (Figure 3).

Voriconazole was continued orally or through a feeding tube to avoid the accumulation of cyclodextrin during the intravenous formation of voriconazole due to renal impairment, and was temporarily replaced by intravenous fos-fluconazole. Although therapeutic drug monitoring was performed, the serum concentration of voriconazole was unstable, ranging from <0.30 to 8.7 μg/mL. Blood culture was negative for fungi on day 22, three days after starting antifungals. However, elevated β-D-glucan (70 and 68 pg/mL on days 75 and 105, respectively) and erythrocyte sedimentation rate (57 and 43 mm/hour on days 82 and 105, respectively) persisted. The vegetation of the mitral valve resolved on day 27. Instead, a new vegetation-like mobile mass on the right coronary cusp of the aortic valve was observed (right two panels of Figure 2). It remained detectable on day 40 and disappeared by day 82. Despite long-term antifungal and antibacterial treatments, tube feeding, and red cell transfusion for anemia of chronic diseases, the patient’s general condition deteriorated due to malnutrition and frailty. Complicated by several other conditions, including a varicella-zoster infection and an allergic response to sulfamethoxazole-trimethoprim, the patient died of aspiration pneumonia on day 119. Complication by *T. asahii* fungemia, after resolution of the initial bacteremia, presumably led to the patient's debilitation and ultimately to their death.

## Discussion

Invasive infections caused by *Trichosporon* spp. are increasing, particularly in immunosuppressed patients, with high mortality [7]. However, the routes of trichosporonosis have not been fully investigated. Bloodstream infection through intravenous devices, invasion through damaged skin barriers, and fungal translocation from the gastrointestinal tract through damaged mucosal tissues are suspected to be important causes of trichosporonosis. Our patient had no placement of central venous catheters, and no skin lesions were observed. Although peripheral line-associated bloodstream infections are reported to be an important cause of candidemia in hospitalized patients [13], there is no literature about trichosporonosis to our knowledge. As there were no signs or symptoms of inflammation at the insertion site of the peripheral line in this patient, bloodstream infection through the devices may be less likely.

Tashiro et al. reported erosion or ulcers of the gastrointestinal tract, proliferation, and vascular invasion of *Trichosporon* species in autopsy cases of patients who were undergoing chemotherapy for hematological malignancies and had a history of antimicrobial use, suggesting that mucosal damage due to anticancer agents combined with disturbance of the gut microbiota due to antimicrobial use are primary factors leading to invasion of *Trichosporon* species from the gut into the systemic bloodstream [11]. Our patient had a history of colon cancer that was managed with surgery and no signs of recurrence. However, polymicrobial bacteremia before *T. asahii* fungemia caused by *E. cloacae*, *K. oxytoca*, *E. faecalis*, and MRSA suggested bacterial invasion through the damaged gut mucosa. Of note, antibacterial use may induce changes in the microbial flora. A disturbed gut flora due to antimicrobial treatment probably led to damaged intestinal mucosal tissues that facilitated the invasion of *T. asahii* into the bloodstream.

Diabetes mellitus is associated with trichosporonosis in patients with hematological disorders [10]. Trichosporonosis can occur via lower limb wounds. Moreover, non-neutropenic diabetic patients are at risk, and the infection does not necessarily originate from skin lesions [14]. Our patient was not neutropenic and had no current malignancy, no administration of immunosuppressive agents or antifungals, no intensive care unit (ICU) admission, and no central venous catheters. His presentation at admission, including hypoalbuminemia, bilateral pleural effusion, ascites, and subcutaneous edema, suggested severe malnutrition. Multiple factors, including age, diabetes, and critical conditions due to polymicrobial bacteremia, presumably contribute to the development of *T. asahii* fungemia, infectious endocarditis, and osteomyelitis. One limitation of this case was that the pathogenesis of endocarditis and osteomyelitis was not confirmed microbiologically. It is not known which species detected in blood culture (*E. cloacae*, *K. oxytoca*, *E. faecalis*, MRSA, or *T. asahii*) caused these conditions. However, the presence of these critical conditions suggested prolonged bacterial and/or *T. asahii* infection, warranting long-term continuation of antibacterials and voriconazole.

Cases of invasive trichosporonosis in non-neutropenic patients are relatively rare [15-17]. In our hospital, a secondary care facility in a super-aged society (more than 30% of the population is aged 65 years or older) without an ICU or sterile ward, this is the only case of fungemia caused by *Trichosporon* species in the past seven years. However, following COVID-19, we did have a fatal case of disseminated cryptococcal infection with positive blood cultures in an elderly patient who was on corticosteroid therapy [18]. Aging of the population, increased exposure to medical procedures, and critical conditions associated with COVID-19 in recent years [19, 20] have presumably led to diverse backgrounds and clinical manifestations of trichosporonosis. With no nationwide surveillance, it is not possible to know if the incidence of trichosporonosis increased after the COVID-19 pandemic. Considering that not only infectious disease specialists and hemato-oncologists, but also general physicians may encounter trichosporonosis, non-neutropenic invasive trichosporonosis warrants further investigation. Additionally, studies to explore the prevalence, characteristics, and prognosis of invasive non-*Candida* fungal infections, including trichosporonosis, in various populations are necessary.

## Conclusions

This case report described a case of *T. asahii* fungemia following polymicrobial bacteremia in a non-neutropenic elderly patient with diabetes mellitus and severe malnutrition. Although trichosporonosis mostly affects immunocompromised individuals, non-neutropenic cases have emerged. We believe that cases of invasive trichosporonosis in non-neutropenic patients should receive greater attention. Further investigations are urgently required.
